# Full-Genome Analysis of Avian Influenza A(H5N1) Virus from a Human, North America, 2013

**DOI:** 10.3201/eid2005.140164

**Published:** 2014-05

**Authors:** Kanti Pabbaraju, Raymond Tellier, Sallene Wong, Yan Li, Nathalie Bastien, Julian W. Tang, Steven J. Drews, Yunho Jang, C. Todd Davis, Kevin Fonseca, Graham A. Tipples

**Affiliations:** Provincial Laboratory for Public Health, Calgary, Alberta, Canada (K. Pabbaraju, R. Tellier, S. Wong, J.W. Tang, S.J. Drews, K. Fonseca, G.A. Tipples);; Public Health Agency of Canada, Winnipeg, Manitoba, Canada (Y. Li, N. Bastien);; Centers for Disease Control and Prevention, Atlanta, Georgia, USA (Y. Jang, C.T. Davis);; University of Calgary, Calgary (R. Tellier, K. Fonseca, S.J. Drews);; University of Manitoba, Winnipeg (Y. Li); and University of Alberta, Edmonton, Alberta, Canada (J.W. Tang, G.A. Tipples)

**Keywords:** avian influenza, H5N1 subtype, highly pathogenic avian influenza, HPAI, North America, reassortant, influenza A, full genome, human, H9N2 subtype, viruses, influenza, genome analysis, Canada

## Abstract

Full-genome analysis was conducted on the first isolate of a highly pathogenic avian influenza A(H5N1) virus from a human in North America. The virus has a hemagglutinin gene of clade 2.3.2.1c and is a reassortant with an H9N2 subtype lineage polymerase basic 2 gene. No mutations conferring resistance to adamantanes or neuraminidase inhibitors were found.

Since the 1997 emergence of highly pathogenic avian influenza (HPAI) A(H5N1) virus in Hong Kong, China, 648 HPAI A(H5N1) infections and 384 associated deaths in humans have been reported. During 2013, Cambodia reported the most human infections, followed by Egypt, Indonesia, China, and Vietnam (www.who.int/influenza/human_animal_interface/H5N1_cumulative_table_archives/en/, December 10, 2013, report). In December 2013, an HPAI A(H5N1) infection was reported in a Canadian resident who recently returned from China. No human or poultry HPAI A(H5N1) infections had been previously reported in North America.

## Case Report and Laboratory Investigations

Preliminary details of this case have been reported (*1*) (Technical Appendix 1). The patient initially sought care for respiratory symptoms; however, the probable cause of death was listed as meningoencephalitis, an unusual outcome for HPAI A(H5N1) infections in humans. Detailed interviews with close contacts have not identified exposure to infected avian sources or environmental contamination, although these investigations are continuing. Because symptom onset occurred during a return flight from China, it is probable that the patient was exposed to the virus while in China.

Nasopharyngeal swab (NP) samples, bronchoalveolar lavage (BAL), and cerebrospinal fluid (CSF) samples tested positive for influenza A(H5N1) virus by various molecular testing methods, including sequencing, at the Provincial Laboratory for Public Health and the National Microbiology Laboratory, Public Health Agency of Canada (*1*). An isolate cultured from BAL (A/Alberta/01/2014) underwent full-genome sequencing (methods available in online Technical Appendix 1); analysis results are presented here.

Partial sequences of virus from the primary specimens (shown in parentheses) included 1,378 bp of the hemagglutinin (HA) gene (CSF, BAL, NP), 1,350 bp of the neuraminidase gene (BAL), 810 bp of the matrix gene (NP), and 687 bp of the polymerase basic 2 (PB2) gene (NP). These sequences were identical to corresponding sequences obtained from the isolate, suggesting the absence of cell culture–induced changes.

**Figure 1:**
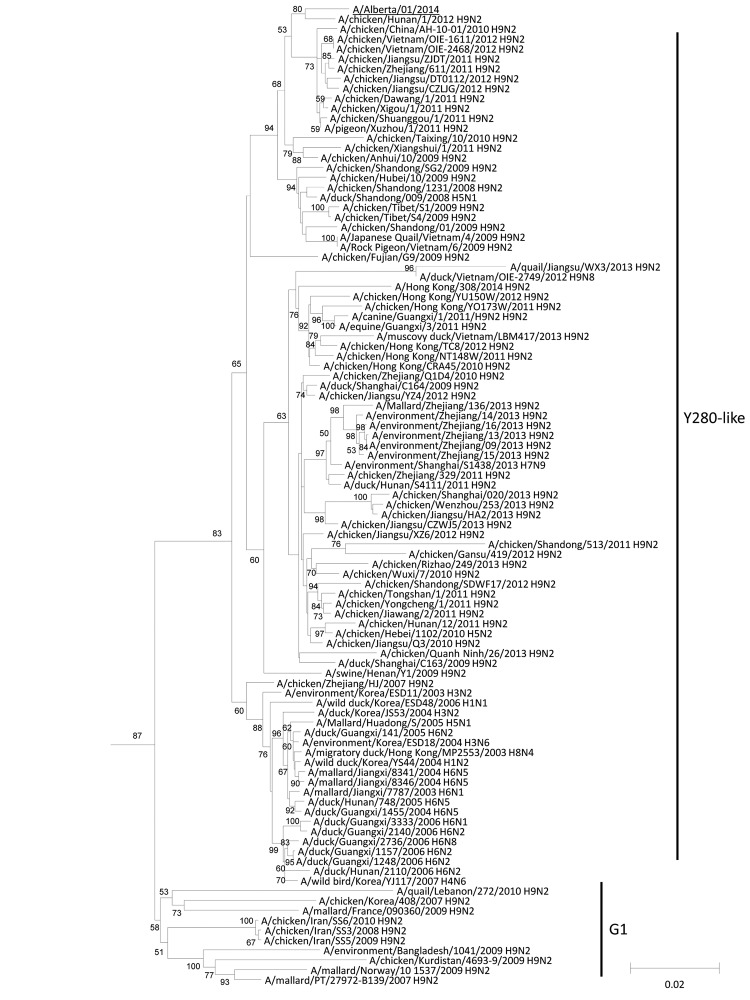
Neighbor-joining phylogenetic tree of the polymerase basic 2 (PB2) genes of H9N2 subtype lineage avian influenza A viruses with A/Alberta/01/2014 (GISAID accession no. EPI500778). The avian influenza A(H5N1) virus detected in Canada is underlined. Major lineages of the H9N2 subtype–like PB2 genes are depicted to the right of the phylogenetic clusters. Bootstraps generated from 1,000 replicates are shown at branch nodes. Scale bar represents nucleotide substitutions per site. GSAID, Global Initiative on Sharing Avian Influenza Data.

**Figure 2:**
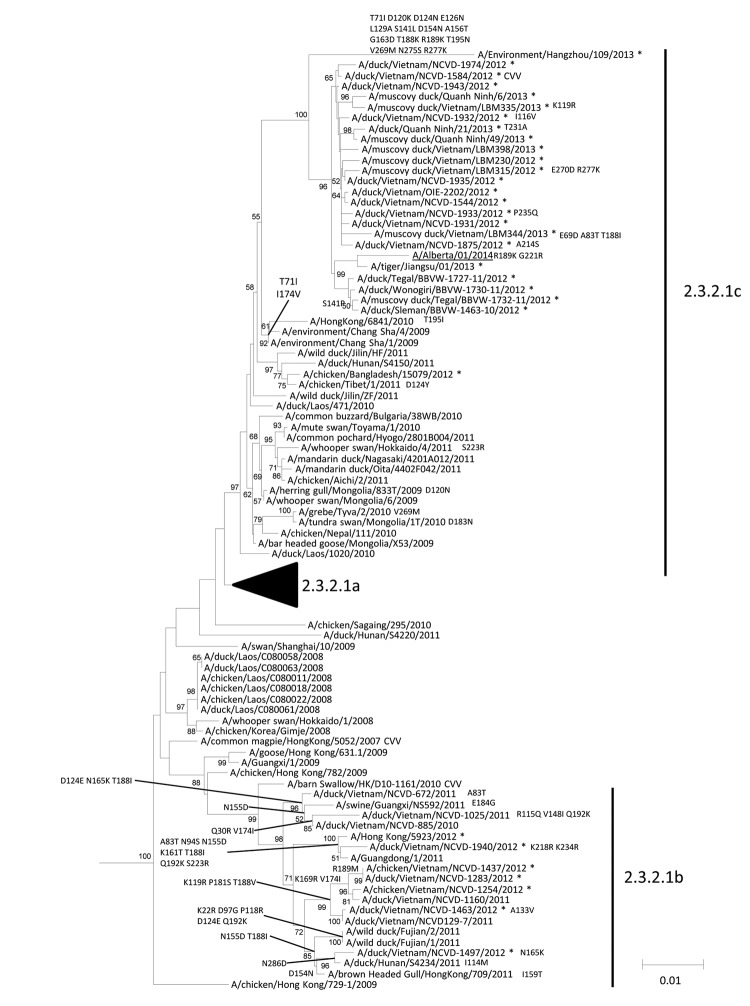
Neighbor-joining phylogenetic tree of the hemagglutinin (HA) genes of clade 2.3.2.1 highly pathogenic avian influenza A(H5N1) viruses with A/Alberta/01/2014 (GISAID accession no. EPI500771). The avian influenza A(H5N1) virus detected in Canada is underlined. The nearest reassortant World Health Organization candidate vaccine viruses (CVV) for each group of clade 2.3.2.1 are denoted by CVV. Asterisks indicated viruses collected in 2012–2014. Amino acid differences at branch nodes indicate HA1 substitutions relative to the nearest CVV for clade 2.3.2.1 viruses (group 2.3.2.1c, A/duck/Vietnam/NCVD-1584/2012; group 2.3.2.1b, A/barn-swallow/HK/D10–1161/2010). Mutations to the right of each strain name indicate amino acid changes found only in that virus relative to the nearest CVV. Bootstraps generated from 1,000 replicates are shown at branch nodes. Scale bar represents nucleotide substitutions per site. Black arrowhead indicates position of clade 2.3.2.1a. GSAID, Global Initiative on Sharing Avian Influenza Data.

BLAST (http://blast.ncbi.nlm.nih.gov/Blast.cgi) analysis of each gene of A/Alberta/01/2014 showed that 7 of 8 genes shared ≥99% identity at the nucleotide and protein levels with HPAI A(H5N1) viruses of avian origin. However, the PB2 gene showed 98% nt similarity and 99% aa identity to avian influenza A(H9N2) viruses collected in China. Phylogenetic analysis of each gene (Technical Appendix 2) with sequences from related viruses confirmed that only the PB2 gene resulted from reassortment with an avian influenza A virus containing an H9N2 subtype lineage PB2 gene (Figure 1). Phylogenetic analysis of the HA gene demonstrated that the virus belongs to clade 2.3.2.1c (*2*) (Figure 2), which has been detected in many countries and has recently been reported in China, Vietnam, and Indonesia (*2*). The HA gene of A/Alberta/01/2014 (H5N1) was most closely related to the sequence an HPAI A(H5N1) virus from a tiger that died in 2013 at a zoo in Jiangsu, China. This combination of clade 2.3.2.1c lineage HA, neuraminidase, and internal gene segments derived from influenza A(H5N1) viruses and an H9N2 subtype lineage PB2 gene indicated that this virus is a previously undescribed genotype of HPAI A(H5N1).

To assess the virus for molecular markers of pandemic risk, we reviewed all protein sequences for mutations listed in the H5N1 Genetic Changes Inventory (*3*).The HA protein possessed a multibasic amino acid cleavage site motif (PQRERRRKR*G) similar to other clade 2.3.2.1 viruses (*4*). The sequence of the 220-loop receptor binding site (RBS) contained the typical avian amino acids, Q222/G224, predictive of a preference for the avian α2,3 rather than the human α2,6 sialic acid (SA) host cell receptor (*5*); all HA gene numbering is based on H5 viruses unless otherwise indicated. The RBS sequence was identical in the NP and BAL samples, suggesting the absence of adaptive changes in the cultured isolate. The G221R substitution, uncommon in HPAI A(H5N1) virus, was detected in the RBS. Previously reported in a clade 2 HPAI A(H5N1) virus (GenBank accession no. ABR13964), R221 has been shown in influenza A/H3 (R225 by H3 numbering) to slightly increase binding to human erythrocytes (*6*). Other mutations of interest in A/Alberta/01/2014 were D94N, S133A, S155N, and T156A. D94N decreased binding to α2,3 SA and increased it to α2,6 SA in a pseudotyping assay (*7*). S133A, together with T188I (not present in A/Alberta/01/2014), increased binding to α2,6 SA by pseudotyping and glycan array assays (*8*). When together, S155N and T156A also increased binding to α2,6 SA (assayed with resialated erythrocytes). T156A abrogates a *N*-glycosylation site and, when together with S223N (not found in A/Alberta/01/2014), may improve virus replication in the upper respiratory tract of ferrets (*9*); T156A is consistently found in ferret-adapted mutants capable of airborne transmission (*5*). The collective effects of all these mutations and their phenotypic manifestations are unclear.

Comparison of the HA amino acid sequence of A/Alberta/01/2014 with that of the nearest H5N1 clade 2.3.2.1c World Health Organization candidate vaccine virus (A/duck/Vietnam/NCVD-1584/2012) identified only 2 aa substitutions in the HA1 region, R189K and G221R. Although position 189 was within the putative antigenic site B, the overall conservation of sequence suggests that A/Alberta/01/2014 is a close antigenic match to the candidate vaccine virus.

In agreement with Xu et al. (*4*), no mutations conferring reduced susceptibility to neuraminidase inhibitors were identified for clade 2.3.2.1. The predicted amino acid sequence of the M2 protein did not reveal any changes associated with reduced susceptibility to adamantanes (*10*). Mutation V27I was found, but its significance is uncertain. Mutations N30D and T215A found in the M1 gene of A/Alberta/01/2014 were associated with increased virulence in mice. The cumulative effect of these changes may result in increased lethality (*11*).

The PB2 sequence showed the presence of E627 in both the primary specimen and isolate, establishing the lack of a well-known mammalian adaptation motif (*5*,*12*). Amino acid changes L89V, G309D, T339K, R477G, I495V, and K627E and a change to Met at the predicted position A676T (*13*) were noted in the A/Alberta/01/2014 isolate. These PB2 substitutions in conjunction with changes in the M1 and HA proteins (only some of which were identified) have been described to enhance polymerase activity and virulence in mice. Experiments in mice also demonstrated that compensatory amino acid substitutions in PB2 can rescue polymerase activity in K627E mutants (*13*). Lethal HPAI A(H5N1) isolates, such as A/quail/Vietnam/36/04, show the presence of E627, suggesting that compensatory mutations are possible in PB2 and other genes (*14*). The PB1 protein showed the P598L mutation reported to enhance polymerase activity in mammalian cells and mice (*3*). This change has been reported to enhance the polymerase activity of an attenuated human virus carrying the PB2 K627E mutation (*15*). Of the polymerase mutations hypothesized to increase the RNA polymerase activity of HPAI A(H5N1) viruses, namely P149S, R226H, K357I, and T515S, only two, 149S and 357T, were present in the A/Alberta/01/2014 isolate (*3*).

Mutations in the nucleoprotein gene reported to enhance replication efficiency, virulence, and transmission (*3*) were absent in the isolate. Several NS1 mutations reported to increase virulence in mice were present in A/Alberta/01/2014: P42S, D87E, L98F, and I101M; a 4-bp deletion from nt 80–84, along with the D92E shift; and the PDZ ligand domain (ESEV) at the C terminus (*3*). The multifunctional NS1 protein is a recognized virulence determinant that counters the cellular innate immune response, and the P42S change has been shown to antagonize interferon induction and prevent activation of the nuclear factor–κB and interferon regulatory factor–3 pathways (*16*).

## Conclusion

Analysis of the whole genome of HPAI A(H5N1) virus provides valuable insight into the presence of mutations that may reflect adaptive changes, altered virulence, and/or transmission phenotype. Because of the unique manifestation of neurologic symptoms and encephalitis reported in this patient, additional studies are needed to understand the broader aspects of virus heterogeneity and its role in this fatal case.

Technical Appendix 1 Case details and methods for a full-genome analysis of highly pathogenic avian influenza A(H5N1) virus isolated from clinical samples of an infected human, North America, 2013.

Technical Appendix 2 Neighbor-joining phylogenetic trees of the polymerase basic 1, polymerase, nucleoprotein, neuraminidase, matrix, and nonstructural protein genes of highly pathogenic avian influenza A(H5N1) viruses with A/Alberta/01/2014.
