# Supplementary material for: Full-Genome Analysis of Avian Influenza A(H5N1) Virus from a Human, North America, 2013
Source: Emerg Infect Dis. 2014 May;20(5):887–91. doi: 10.3201/eid2005.140164 (PMC4012823; doi:10.3201/eid2005.140164)
Supplement: Technical Appendix 1 — Case details and methods for a full-genome analysis of highly pathogenic avian influenza A(H5N1) virus isolated from clinical samples of an infected human, North America, 2013. [file 14-0164-Techapp-s1.pdf]

Article DOI: http://dx.doi.org/10.3201/eid2005.140164

Full-Genome Analysis of Avian Influenza A(H5N1) Virus from a Human, North America, 2013

Technical Appendix 1

Case Details

The individual, a young adult accompanied by a family member, left Canada for China on December 6, 2013, and was exclusively in Beijing, in urban locations. There was no reported contact with live poultry, visits to wet markets or handling of fresh poultry.

During the return flight on December 27, 2013, the individual experienced symptoms of malaise, chest pain, and fever and presented to the local Emergency Department on December 28, 2013. The complete blood count (CBC) showed a total white blood count (wbc) of 12.6×10^9 cells/L (reference range 4.0–10.0 $\times 10^9$ cells/L) with raised neutrophils (11.1×10^9 cells/L) and low lymphocytes (0.8×10^9 cells/L). A chest x-ray and CT scan revealed a right apical infiltrate; a diagnosis of pneumonia was made, the patient was prescribed levofloxacin and discharged home.

The individual returned to the same Emergency Department on January 01, 2014, now with worsening pleuritic chest pains and shortness of breath, a mild headache, exacerbated by head movement, right upper quadrant and epigastric pain, nausea and vomiting with no diarrhea. A chest x-ray showed a multi-lobar pneumonia, with moderate effusion, reflecting significant progression when compared with the x-ray from the first ED visit. A thoracentesis, performed while in the ED, revealed a dark amber cloudy fluid that was sterile in bacterial culture. The CBC again showed a wbc count of 10.2×10^9 cells/L, neutrophil count of 9.5×10^9 cells/L, platelet count within the normal range, normal ALT, slightly elevated AST at 46U/L (reference range 7–40U/L) and LDH at 288U/L (reference range 100–225U/L).

Admission to a general medicine ward for investigation was facilitated and treatment was initiated with intravenous piperacillin-tazobactam. On January 2, 2014 the individual reported visual changes and on-going headache, and coupled with increasing oxygen requirements was admitted to the ICU for intubation and ventilation. Early in the morning of January 3rd, the individual developed a sudden episode of tachycardia and severe hypertension followed by hypotension requiring inotropic support. At this stage, pupils were dilated and there was no response to pain.

A CT brain suggested diffuse encephalitis and intracranial hypertension; the neurologic examination was consistent with brain death. A MRI/MRA showed significant generalized edema, evidence of meningitis and ventriculitis and significant reduction in cerebral blood flow. A lumbar puncture was performed after brain death determination and before removal of ventilatory and inotropic support.

Methods

Samples

Nasopharyngeal swabs (NP) and broncho-alveolar lavage (BAL) were sent to the Provincial Laboratory for investigation of influenza and other respiratory viral agents. The cerebrospinal fluid (CSF) was sent for testing of the herpesvirus group, enterovirus and parechoviruses. Two NP swabs, CSF and BAL initially tested positive for influenza A by RT-PCR targeting the M gene, subsequently determined to be H5 subtype by real-time RT-PCR with viral titers conducive for direct sequencing (*1*). Sequence data was obtained directly from the patient samples and compared to the cultured isolate for the following specimen types and gene targets: Matrix from the NP, neuraminidase from the BAL, hemagglutinin from the CSF, BAL and NP and polymerase B2 subunit from the NP based on availability of patient specimen and nucleic acid extracts.

Our preliminary findings were subsequently confirmed by the National Microbiology Laboratory (NML) that this strain was an avian influenza A(H5N1).

Isolation of influenza A H5N1 in cell culture

For virus propagation, 10 μL of the BAL specimen was inoculated into the Madin-Darby canine kidney cell line (MDCK) (CCL34, American Type Culture Collection, Rockville, Md.)

and examined for cytopathic effect. The MDCK cells were maintained in Eagles's MEM containing 100U/mL Penicillin, 100 μg/mL Streptomycin, 0.292 mg/mL Glutamine, 25mM Hepes buffer, 0.1 mM MEM nonessential amino acid, 1 mM MEM sodium pyruvate and 2 μg/mL TCPK-trypsin. Cells were incubated at 37°C in a CO_2 incubator and observed daily for cytopathic effect (World Health Organization. Global Influenza Surveillance Network.Manual for the laboratory diagnosis and virological surveillance of influenza.2011. http://whqlibdoc.who.int/publications/2011/9789241548090_eng.pdf). Influenza A(H5N1) virus was confirmed by real-time RT-PCR. The isolate was designated as A/Alberta/01/2014.

Full genome Sanger sequencing from the BAL isolate

Reactions were performed using BigDye-Terminator v3.1 Cycle Sequencing Reaction Kit on a 3730 xl Genetic Analyzer (Applied Biosystems, Foster City, CA, USA) following the manufacturer's instructions. The A/Alberta/01/2014 virus was bi-directionally sequenced across all segments using a combination of in-house and a universal primer sets for the full-length amplification of the genome (*1*). Sequences were assembled, curated, and edited using Sequencher 5.2.3 (Gene Codes Corporation, Ann Arbor, MI, USA). All sequences have been submitted to the Global Initiative on Sharing Avian Influenza Data (GISAID) (PB2: EPI500778; PB1: EPI500777; PA: EPI500776; HA: EPI500771; NP: EPI500774; NA: EPI500773; MP: EPI500772; NS: EPI500775).

Partial Sanger sequencing directly from specimen

All amplifications were performed using the QiagenOneStep RT-PCR kit (Qiagen, Mississauga, Ontario, Canada). Bi-directional Sanger sequencing was performed using the BigDye® Terminator v3.1 Cycle Sequencing Kit and the BigDyeXTerminator® Purification kit (Life Technologies, Burlington, Ontario, Canada) on the 3500xl Genetic Analyzer (ABI). Sequence analysis was performed using Seqscape v2.7 and Sequencing Analysis SeqA6 (ABI), sequence alignments were performed using Clustal W (BioEdit). Primers used to amplify the different gene segments from the primary specimen can be provided upon request.

Phylogenetic analysis

Full length sequences of each gene segment were aligned to closely related sequences identified through BLAST searches in publicly available influenza sequence databases and previously generated gene segment alignments. Trees were built with MEGA5 software using

the Neighbor-Joining method based on a maximum composite likelihood model and mid-point rooted. The reliability of the trees was estimated by bootstrap re-sampling analysis with 1,000 replications.

Reference

1. Gall A, Hoffmann B, Harder T, Grund C, Beer M. Universal primer set for amplification and sequencing of HA0 cleavage sites of all influenza A viruses. J Clin Microbiol.2008;46:2561–7. PubMed http://dx.doi.org/10.1128/JCM.00466-08
