# Supplementary material for: Full-Genome Analysis of Avian Influenza A(H5N1) Virus from a Human, North America, 2013
Source: Emerg Infect Dis. 2014 May;20(5):887–91. doi: 10.3201/eid2005.140164 (PMC4012823; doi:10.3201/eid2005.140164)
Supplement: Technical Appendix 2 — Neighbor-joining phylogenetic trees of the polymerase basic 1, polymerase, nucleoprotein, neuraminidase, matrix, and nonstructural protein genes of highly pathogenic avian influenza A(H5N1) viruses with A/Alberta/01/2014. [file 14-0164-Techapp-s2.pdf]

Article DOI: http://dx.doi.org/10.3201/eid2005.140164

Full-Genome Analysis of Avian Influenza A(H5N1) Virus from a Human, North America, 2013

Technical Appendix 2

PB1

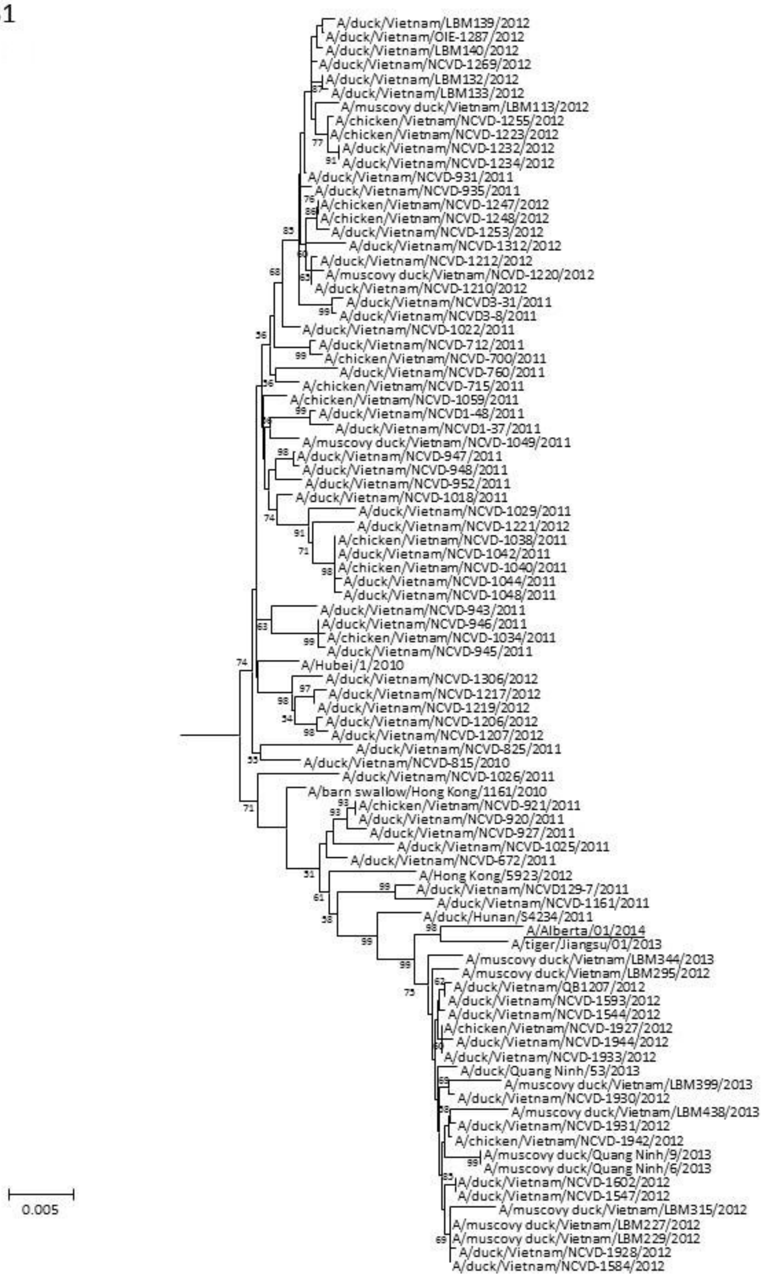

PA

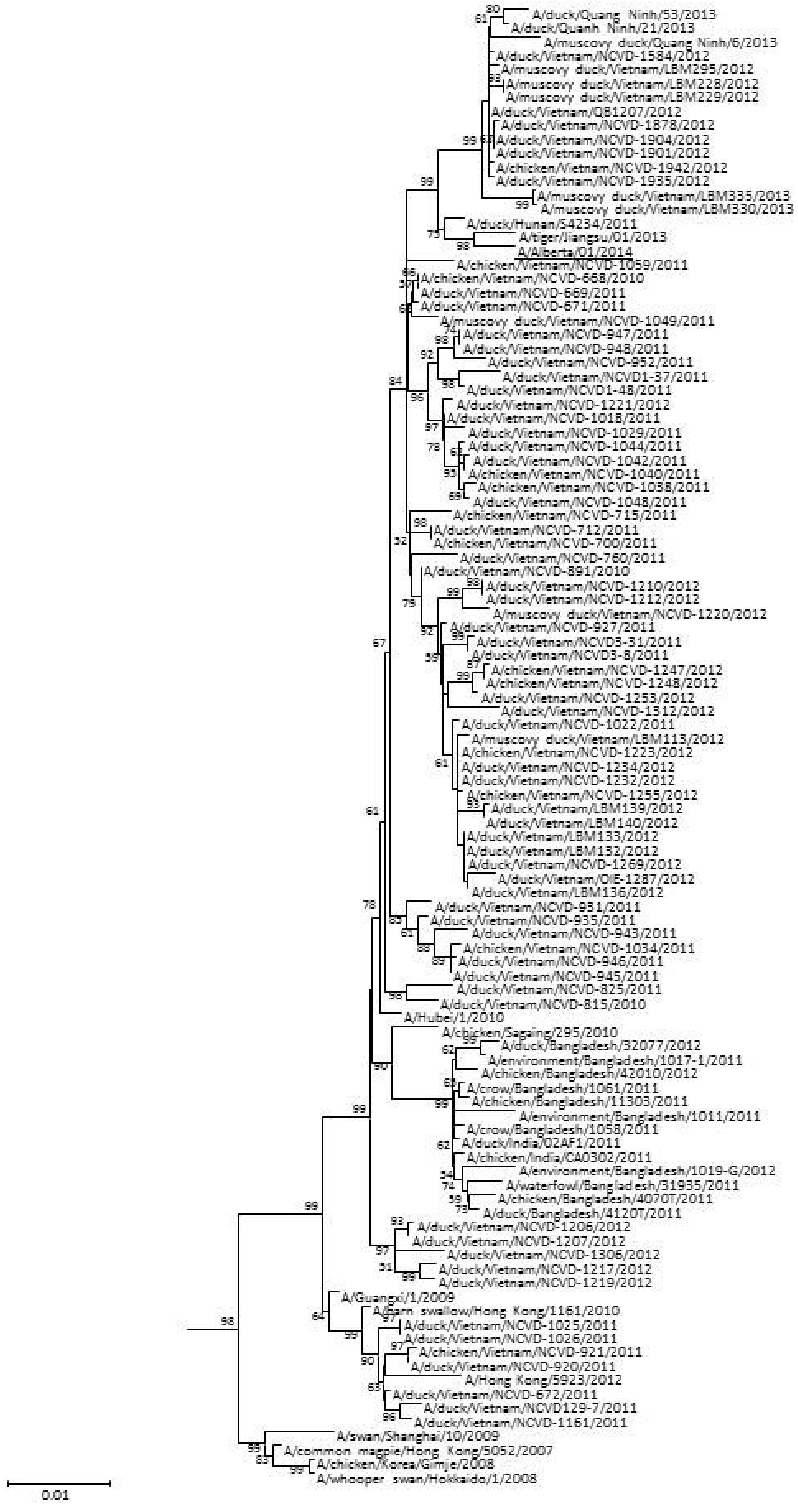

NP

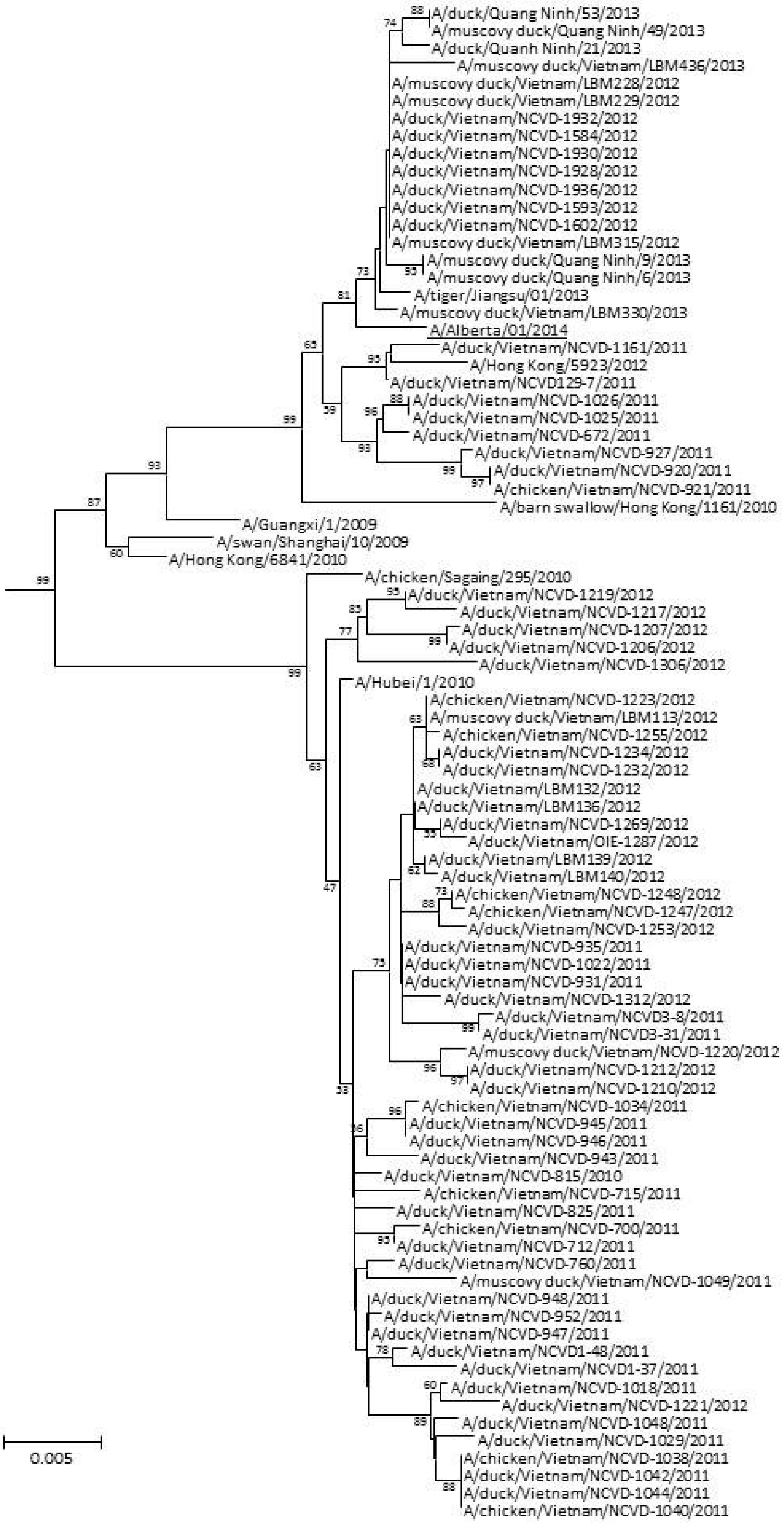

A/duck/Quang Ninh/53/2013
A/muscovy duck/Quang Ninh/49/2013
A/duck/Quanh Ninh/21/2013
A/muscovy duck/Vietnam/LBM436/2013
A/muscovy duck/Vietnam/LBM228/2012
A/muscovy duck/Vietnam/LBM229/2012
A/duck/Vietnam/NCVD-1932/2012
A/duck/Vietnam/NCVD-1584/2012
A/duck/Vietnam/NCVD-1930/2012
A/duck/Vietnam/NCVD-1928/2012
A/duck/Vietnam/NCVD-1936/2012
A/duck/Vietnam/NCVD-1593/2012
A/duck/Vietnam/NCVD-1602/2012
A/muscovy duck/Vietnam/LBM315/2012
A/muscovy duck/Quang Ninh/9/2013
A/muscovy duck/Quang Ninh/6/2013
A/tiger/Jiangsu/01/2013
A/muscovy duck/Vietnam/LBM330/2013
A/Alberta/01/2014
A/duck/Vietnam/NCVD-1161/2011
A/Hong Kong/5923/2012
A/duck/Vietnam/NCVD129-7/2011
A/duck/Vietnam/NCVD-1026/2011
A/duck/Vietnam/NCVD-1025/2011
A/duck/Vietnam/NCVD-672/2011
A/duck/Vietnam/NCVD-927/2011
A/duck/Vietnam/NCVD-920/2011
A/chicken/Vietnam/NCVD-921/2011
A/barn swallow/Hong Kong/1161/2010
A/Guangxi/1/2009
A/swan/Shanghai/10/2009
A/Hong Kong/6841/2010
A/chicken/Sagaing/295/2010
A/duck/Vietnam/NCVD-1219/2012
A/duck/Vietnam/NCVD-1217/2012
A/duck/Vietnam/NCVD-1207/2012
A/duck/Vietnam/NCVD-1206/2012
A/duck/Vietnam/NCVD-1306/2012
A/Hubei/1/2010
A/chicken/Vietnam/NCVD-1223/2012
A/muscovy duck/Vietnam/LBM113/2012
A/chicken/Vietnam/NCVD-1255/2012
A/duck/Vietnam/NCVD-1234/2012
A/duck/Vietnam/NCVD-1232/2012
A/duck/Vietnam/LBM132/2012
A/duck/Vietnam/LBM136/2012
A/duck/Vietnam/NCVD-1269/2012
A/duck/Vietnam/OIE-1287/2012
A/duck/Vietnam/LBM139/2012
A/duck/Vietnam/LBM140/2012
A/chicken/Vietnam/NCVD-1248/2012
A/chicken/Vietnam/NCVD-1247/2012
A/duck/Vietnam/NCVD-1253/2012
A/duck/Vietnam/NCVD-935/2011
A/duck/Vietnam/NCVD-1022/2011
A/duck/Vietnam/NCVD-931/2011
A/duck/Vietnam/NCVD-1312/2012
A/duck/Vietnam/NCVD3-8/2011
A/duck/Vietnam/NCVD3-31/2011
A/muscovy duck/Vietnam/NCVD-1220/2012
A/duck/Vietnam/NCVD-1212/2012
A/duck/Vietnam/NCVD-1210/2012
A/chicken/Vietnam/NCVD-1034/2011
A/duck/Vietnam/NCVD-945/2011
A/duck/Vietnam/NCVD-946/2011
A/duck/Vietnam/NCVD-943/2011
A/duck/Vietnam/NCVD-815/2010
A/chicken/Vietnam/NCVD-715/2011
A/duck/Vietnam/NCVD-825/2011
A/chicken/Vietnam/NCVD-700/2011
A/duck/Vietnam/NCVD-712/2011
A/duck/Vietnam/NCVD-760/2011
A/muscovy duck/Vietnam/NCVD-1049/2011
A/duck/Vietnam/NCVD-948/2011
A/duck/Vietnam/NCVD-952/2011
A/duck/Vietnam/NCVD-947/2011
A/duck/Vietnam/NCVD1-48/2011
A/duck/Vietnam/NCVD1-37/2011
A/duck/Vietnam/NCVD-1018/2011
A/duck/Vietnam/NCVD-1221/2012
A/duck/Vietnam/NCVD-1048/2011
A/duck/Vietnam/NCVD-1029/2011
A/chicken/Vietnam/NCVD-1038/2011
A/duck/Vietnam/NCVD-1042/2011
A/duck/Vietnam/NCVD-1044/2011
A/chicken/Vietnam/NCVD-1040/2011
0.005

NA

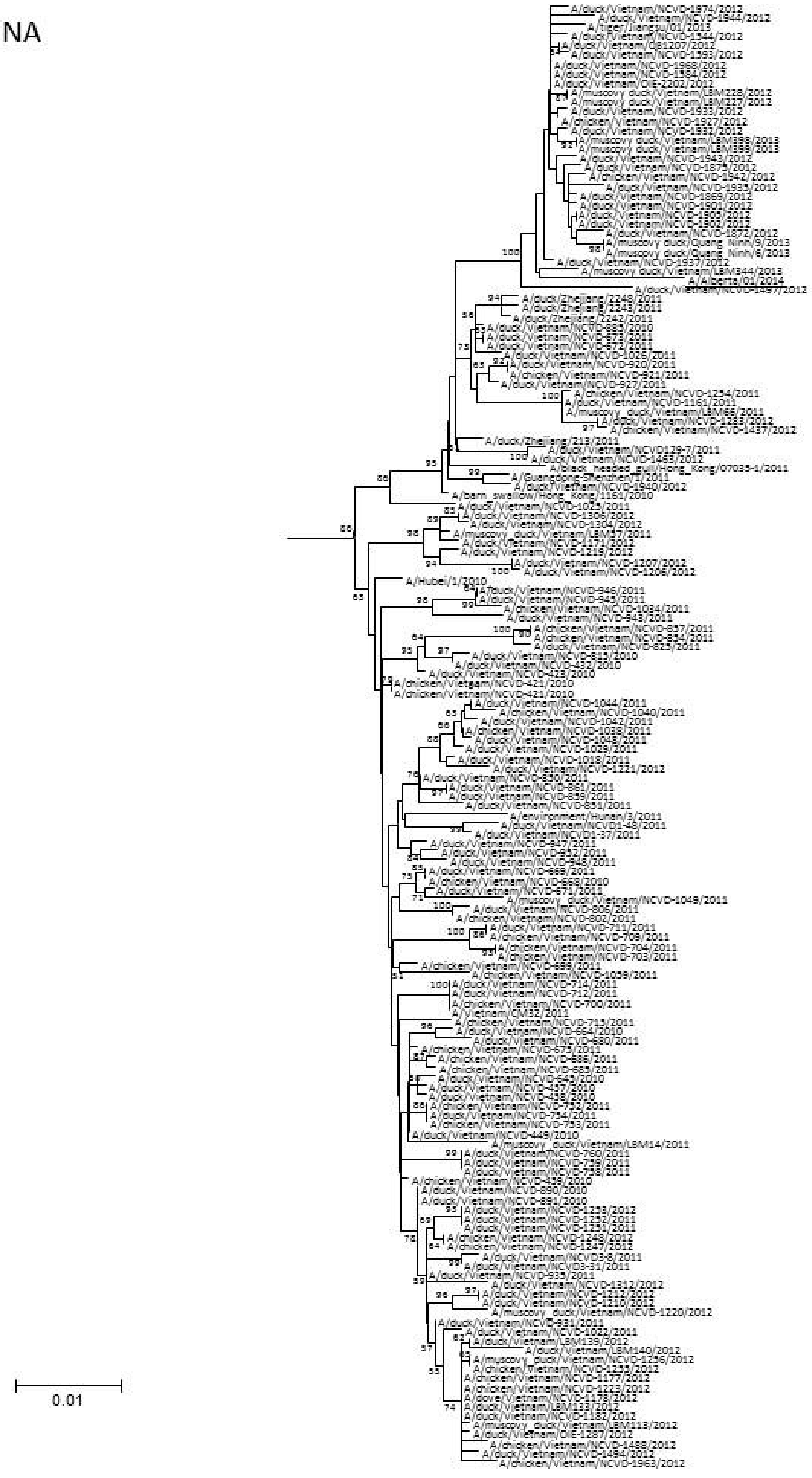

M

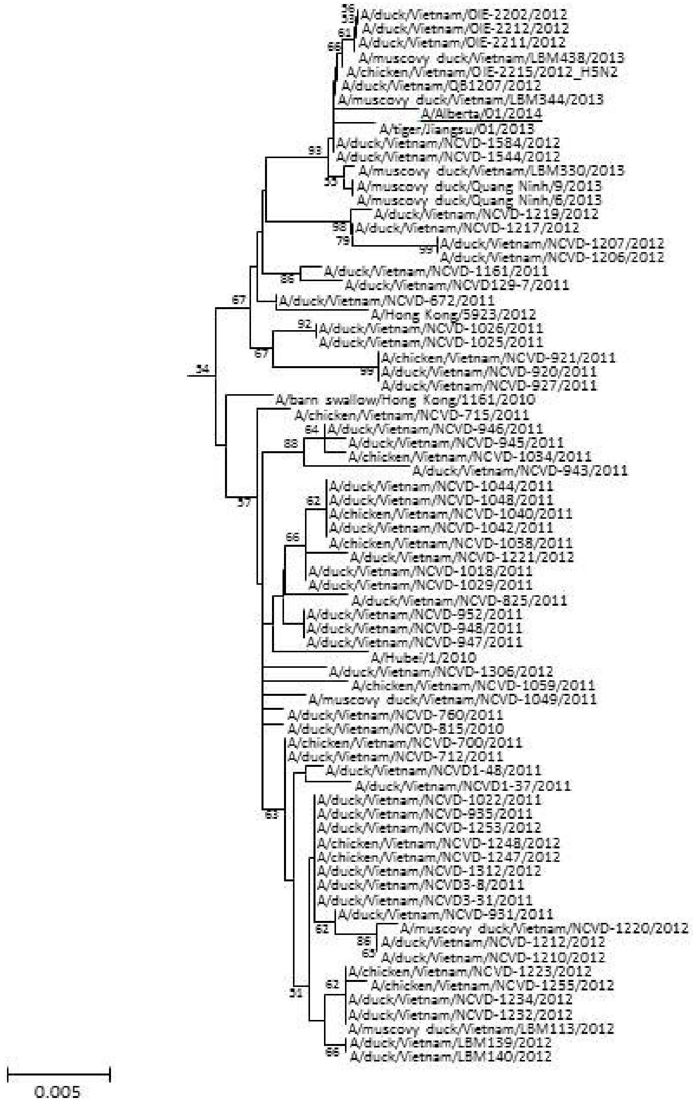
A/duck/Vietnam/OIE-2202/2012
A/duck/Vietnam/OIE-2212/2012
A/duck/Vietnam/OIE-2211/2012
A/muscovy duck/Vietnam/LBM438/2013
A/chicken/Vietnam/OIE-2215/2012_H5N2
A/duck/Vietnam/QB1207/2012
A/muscovy duck/Vietnam/LBM344/2013
A/Alberta/01/2014
A/tiger/Jiangsu/01/2013
A/duck/Vietnam/NCVD-1584/2012
A/duck/Vietnam/NCVD-1544/2012
A/muscovy duck/Vietnam/LBM330/2013
A/muscovy duck/Quang Ninh/9/2013
A/muscovy duck/Quang Ninh/6/2013
A/duck/Vietnam/NCVD-1219/2012
A/duck/Vietnam/NCVD-1217/2012
A/duck/Vietnam/NCVD-1207/2012
A/duck/Vietnam/NCVD-1206/2012
A/duck/Vietnam/NCVD-1161/2011
A/duck/Vietnam/NCVD129-7/2011
A/duck/Vietnam/NCVD-672/2011
A/Hong Kong/5923/2012
A/duck/Vietnam/NCVD-1026/2011
A/duck/Vietnam/NCVD-1025/2011
A/chicken/Vietnam/NCVD-921/2011
A/duck/Vietnam/NCVD-920/2011
A/duck/Vietnam/NCVD-927/2011
A/barn swallow/Hong Kong/1161/2010
A/chicken/Vietnam/NCVD-715/2011
A/duck/Vietnam/NCVD-946/2011
A/duck/Vietnam/NCVD-945/2011
A/chicken/Vietnam/NCVD-1034/2011
A/duck/Vietnam/NCVD-943/2011
A/duck/Vietnam/NCVD-1044/2011
A/duck/Vietnam/NCVD-1048/2011
A/chicken/Vietnam/NCVD-1040/2011
A/duck/Vietnam/NCVD-1042/2011
A/chicken/Vietnam/NCVD-1038/2011
A/duck/Vietnam/NCVD-1221/2012
A/duck/Vietnam/NCVD-1018/2011
A/duck/Vietnam/NCVD-1029/2011
A/duck/Vietnam/NCVD-825/2011
A/duck/Vietnam/NCVD-952/2011
A/duck/Vietnam/NCVD-948/2011
A/duck/Vietnam/NCVD-947/2011
A/Hubei/1/2010
A/duck/Vietnam/NCVD-1306/2012
A/chicken/Vietnam/NCVD-1059/2011
A/muscovy duck/Vietnam/NCVD-1049/2011
A/duck/Vietnam/NCVD-760/2011
A/duck/Vietnam/NCVD-815/2010
A/chicken/Vietnam/NCVD-700/2011
A/duck/Vietnam/NCVD-712/2011
A/duck/Vietnam/NCVD1-48/2011
A/duck/Vietnam/NCVD1-37/2011
A/duck/Vietnam/NCVD-1022/2011
A/duck/Vietnam/NCVD-935/2011
A/duck/Vietnam/NCVD-1253/2012
A/chicken/Vietnam/NCVD-1248/2012
A/chicken/Vietnam/NCVD-1247/2012
A/duck/Vietnam/NCVD-1312/2012
A/duck/Vietnam/NCVD3-8/2011
A/duck/Vietnam/NCVD3-31/2011
A/duck/Vietnam/NCVD-931/2011
A/muscovy duck/Vietnam/NCVD-1220/2012
A/duck/Vietnam/NCVD-1212/2012
A/duck/Vietnam/NCVD-1210/2012
A/chicken/Vietnam/NCVD-1223/2012
A/chicken/Vietnam/NCVD-1255/2012
A/duck/Vietnam/NCVD-1234/2012
A/duck/Vietnam/NCVD-1232/2012
A/muscovy duck/Vietnam/LBM113/2012
A/duck/Vietnam/LBM139/2012
A/duck/Vietnam/LBM140/2012
0.005

NS

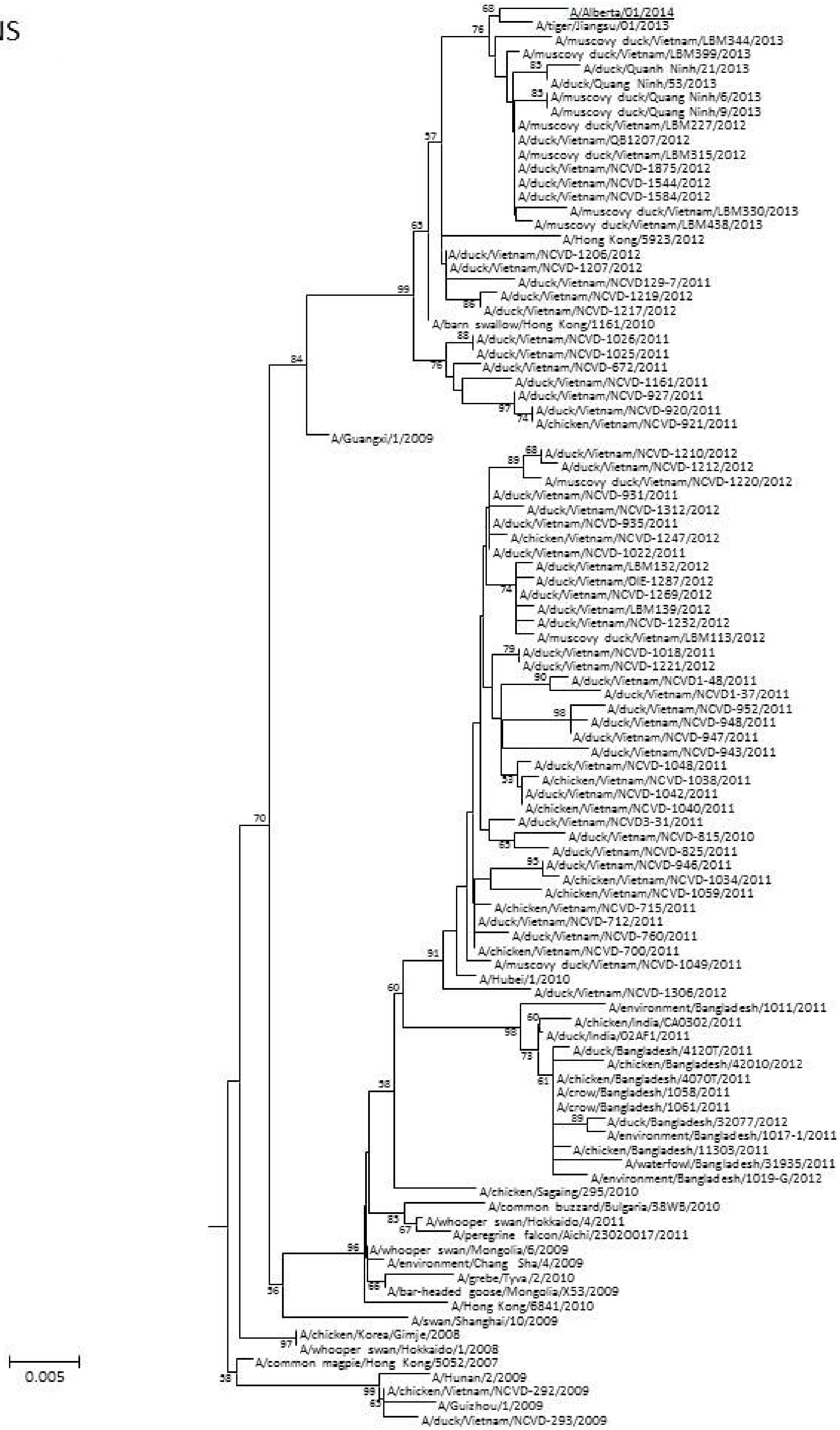
A/Alberta/01/2014
A/tiger/Jiangsu/01/2013
A/muscovy duck/Vietnam/LBM344/2013
A/muscovy duck/Vietnam/LBM399/2013
A/duck/Quanh Ninh/21/2013
A/duck/Quang Ninh/53/2013
A/muscovy duck/Quang Ninh/6/2013
A/muscovy duck/Quang Ninh/9/2013
A/muscovy duck/Vietnam/LBM227/2012
A/duck/Vietnam/QB1207/2012
A/muscovy duck/Vietnam/LBM315/2012
A/duck/Vietnam/NCVD-1875/2012
A/duck/Vietnam/NCVD-1544/2012
A/duck/Vietnam/NCVD-1584/2012
A/muscovy duck/Vietnam/LBM330/2013
A/muscovy duck/Vietnam/LBM438/2013
A/Hong Kong/5923/2012
A/duck/Vietnam/NCVD-1206/2012
A/duck/Vietnam/NCVD-1207/2012
A/duck/Vietnam/NCVD129-7/2011
A/duck/Vietnam/NCVD-1219/2012
A/duck/Vietnam/NCVD-1217/2012
A/barn swallow/Hong Kong/1161/2010
A/duck/Vietnam/NCVD-1026/2011
A/duck/Vietnam/NCVD-1025/2011
A/duck/Vietnam/NCVD-672/2011
A/duck/Vietnam/NCVD-1161/2011
A/duck/Vietnam/NCVD-927/2011
A/duck/Vietnam/NCVD-920/2011
A/chicken/Vietnam/NCVD-921/2011
A/Guangxi/1/2009
A/duck/Vietnam/NCVD-1210/2012
A/duck/Vietnam/NCVD-1212/2012
A/muscovy duck/Vietnam/NCVD-1220/2012
A/duck/Vietnam/NCVD-931/2011
A/duck/Vietnam/NCVD-1312/2012
A/duck/Vietnam/NCVD-935/2011
A/chicken/Vietnam/NCVD-1247/2012
A/duck/Vietnam/NCVD-1022/2011
A/duck/Vietnam/LBM132/2012
A/duck/Vietnam/OIE-1287/2012
A/duck/Vietnam/NCVD-1269/2012
A/duck/Vietnam/LBM139/2012
A/duck/Vietnam/NCVD-1232/2012
A/muscovy duck/Vietnam/LBM113/2012
A/duck/Vietnam/NCVD-1018/2011
A/duck/Vietnam/NCVD-1221/2012
A/duck/Vietnam/NCVD1-48/2011
A/duck/Vietnam/NCVD1-37/2011
A/duck/Vietnam/NCVD-952/2011
A/duck/Vietnam/NCVD-948/2011
A/duck/Vietnam/NCVD-947/2011
A/duck/Vietnam/NCVD-943/2011
A/duck/Vietnam/NCVD-1048/2011
A/chicken/Vietnam/NCVD-1038/2011
A/duck/Vietnam/NCVD-1042/2011
A/chicken/Vietnam/NCVD-1040/2011
A/duck/Vietnam/NCVD3-31/2011
A/duck/Vietnam/NCVD-815/2010
A/duck/Vietnam/NCVD-825/2011
A/duck/Vietnam/NCVD-946/2011
A/chicken/Vietnam/NCVD-1034/2011
A/chicken/Vietnam/NCVD-1059/2011
A/chicken/Vietnam/NCVD-715/2011
A/duck/Vietnam/NCVD-712/2011
A/duck/Vietnam/NCVD-760/2011
A/chicken/Vietnam/NCVD-700/2011
A/muscovy duck/Vietnam/NCVD-1049/2011
A/Hubei/1/2010
A/duck/Vietnam/NCVD-1306/2012
A/environment/Bangladesh/1011/2011
A/chicken/India/CA0302/2011
A/duck/India/02AF1/2011
A/duck/Bangladesh/4120T/2011
A/chicken/Bangladesh/42010/2012
A/chicken/Bangladesh/4070T/2011
A/crow/Bangladesh/1058/2011
A/crow/Bangladesh/1061/2011
A/duck/Bangladesh/32077/2012
A/environment/Bangladesh/1017-1/2011
A/chicken/Bangladesh/11303/2011
A/waterfowl/Bangladesh/31935/2011
A/environment/Bangladesh/1019-G/2012
A/chicken/Sagaing/295/2010
A/common buzzard/Bulgaria/38WB/2010
A/whooper swan/Hokkaido/4/2011
A/peregrine falcon/Aichi/23020017/2011
A/whooper swan/Mongolia/6/2009
A/environment/Chang Sha/4/2009
A/grebe/Tyva/2/2010
A/bar-headed goose/Mongolia/X53/2009
A/Hong Kong/6841/2010
A/swan/Shanghai/10/2009
A/chicken/Korea/Gimje/2008
A/whooper swan/Hokkaido/1/2008
A/common magpie/Hong Kong/5052/2007
A/Hunan/2/2009
A/chicken/Vietnam/NCVD-292/2009
A/Guizhou/1/2009
A/duck/Vietnam/NCVD-293/2009
0.005

Figure. Neighbor-joining phylogenetic trees of the polymerase basic 1 (PB1) (GSAID accession no. EPI500777), polymerase (PA) (GSAID accession no. EPI500776), nucleoprotein (NP) (GSAID accession no. EPI500774), neuraminidase (NA) (GSAID accession no. EPI500773), matrix (M) (GSAID accession no. EPI500772), and nonstructural protein (NS) (GSAID accession no. EPI500775) genes of highly pathogenic avian influenza A(H5N1) viruses with A/Alberta/01/2014. The H5N1 virus detected in Canada is underlined. Bootstraps generated from 1,000 replicates are shown at branch nodes. Scale bar represents nucleotide substitutions per site. GSAID, Global Initiative on Sharing Avian Influenza Data.
